# Treatment gap and mental health service use among Syrian refugees in Sultanbeyli, Istanbul: a cross-sectional survey

**DOI:** 10.1017/S2045796019000660

**Published:** 2019-11-15

**Authors:** D. C. Fuhr, C. Acarturk, M. McGrath, Z. Ilkkursun, E. Sondorp, M. Sijbrandij, P. Ventevogel, P. Cuijpers, M. McKee, B. Roberts

**Affiliations:** 1 Departmentof Health Services Research and Policy, London School of Hygiene and Tropical Medicine, Faculty of Public Health and Policy, London, UK; 2 Department of Psychology, Istanbul Sehir University, Istanbul, Turkey; 3 Department of Health Services Research and Policy, London School of Hygiene and Tropical Medicine, Faculty of Public Health and Policy, KIT Royal Tropical Institute, London, UK; 4 KIT Royal Tropical Institute, Amsterdam, The Netherlands; 5 Department of Clinical, Neuro- and Developmental Psychology, Vrije Universiteit, Amsterdam, The Netherlands; 6 Public Health Section, Division of Programme Management and Support, United High Commissioner for Refugees, Geneva, Switzerland; 7 Department of Health Services Research and Policy, London School of Hygiene and Tropical Medicine, Faculty of Public Health and Policy, London, UK

**Keywords:** Mental health, refugees, Turkey

## Abstract

**Aims:**

Syrian refugees may have increased mental health needs due to the frequent exposure to potentially traumatic events and violence experienced during the flight from their home country, breakdown of supportive social networks and daily life stressors related to refugee life. The aim of this study is to report evidence on mental health needs and access to mental health and psychosocial support (MHPSS) among Syrians refugees living in Sultanbeyli-Istanbul, Turkey.

**Methods:**

A cross-sectional survey was conducted among Syrian refugees aged 18 years or over in Sultanbeyli between February and May 2018. We used random sampling to select respondents by using the registration system of the municipality. Data among 1678 Syrian refugees were collected on mental health outcomes using the Posttraumatic Stress Disorder (PTSD) Checklist (PCL-5) and the Hopkins Symptoms Checklist (HSCL-25) for depression and anxiety. We also collected data on health care utilisation, barriers to seeking and continuing care as well as knowledge and attitudes towards mental health. Descriptive analyses were used.

**Results:**

The estimated prevalence of symptoms of PTSD, depression and anxiety was 19.6, 34.7 and 36.1%, respectively. In total, 249 respondents (15%) screened positive for either PTSD, depression or anxiety in our survey and self-reported emotional/behavioural problems since arriving in Sultanbeyli. The treatment gap (the proportion of these 249 people who did not seek care) was 89% for PTSD, 90% for anxiety and 88% for depression. Several structural and attitudinal barriers for not seeking care were reported, including the cost of mental health care, the belief that time would improve symptoms, fear of being stigmatised and lack of knowledge on where and how to get help. Some negative attitudes towards people with mental health problems were reported by respondents.

**Conclusions:**

Syrian refugees hardly access MHPSS services despite high mental health needs, and despite formally having access to the public mental health system in Turkey. To overcome the treatment gap, MHPSS programmes need to be implemented in the community and need to overcome the barriers to seeking care which were identified in this study. Mental health awareness raising activities should be provided in the community alongside the delivery of psychological interventions. This is to increase help-seeking and to tackle negative attitudes towards mental health and people with mental health problems.

## Introduction

Conflict-affected populations are at an increased risk of mental disorders due to frequent exposure to potentially traumatic events, multiple losses, breakdown of supportive social networks and accumulation of daily life stressors related to refugee life (Miller and Rasmussen, 2010; Steel *et al*., 2009; Silove *et al*., 2017). Recent data of the World Health Organization estimate the age-standardised point prevalence for depression to be 10.8%, for anxiety disorder (including post-traumatic stress disorder (PTSD)) to be 21.7%, and for PTSD alone to be 15.3% at any point in time among all conflict-affected populations (Charlson *et al*., 2019).

Elevated prevalence of symptoms for common mental disorders has also been reported among Syrian refugees who make up the largest single group of displaced people in the world (UNHCR, 2019). Mental disorder symptoms for Syrian refugees living in camps in Turkey and in European host countries have been reported to range from 14 to 44% for depression (Tinghog *et al*., 2017; Acarturk *et al*., 2018; Georgiadou *et al*., 2018; Poole *et al*., 2018), 13 to 31% for anxiety (Tinghog *et al*., 2017; Georgiadou *et al*., 2018) and 11 to 83% for PTSD (Alpak *et al*., 2015; Tinghog *et al*., 2017; Acarturk *et al*., 2018; Cheung *et al*., 2018; Georgiadou *et al*., 2018)

The majority of Syrian refugees live in Turkey. Turkey now hosts an estimated 3.6 million Syrian refugees, which makes it the largest refugee hosting country in the world (UNHCR, 2019). Almost all Syrian refugees live in Turkish host communities in larger cities, and only 7% are living in camps (UNHCR, 2018*a*, 2018*b*). The largest refugee hosting city is Istanbul which accommodates over 500 000 Syrian refugees (UNHCR, 2018*a*, 2018*b*).

Turkey has implemented recent health system reforms to improve access to care for the general population, including Syrian refugees, involving transfer of care into the community (Atun *et al*., 2013), with efforts to integrate mental health into primary health care (Bilge *et al*., 2016; Ekmekci, 2017; IMC, 2017). Syrian refugees who are registered in any Turkish municipality can access services in the public health care system. There is a fee payable for accessing services which is paid for by the government (IMC, 2017). There are also over 100 non-governmental organisations (NGOs) in Turkey which provide mental health and psychosocial support (MHPSS) services to refugees free of charge (IMC, 2017). Barriers faced by Syrian refugees seeking to access the public health care system have been reported and include language barriers and difficulty in navigating a complex foreign health system (Torun *et al*., 2018; UNHCR, 2018*a*). A number of health system innovations have recently been implemented in response to this, including using Syrian medical providers and refugee health centres as platforms of care (WHO, 2017; Akik *et al*., 2019). There are also endeavours to scale up transdiagnostic interventions for Syrian refugees in Turkey such as Problem Management Plus (Sijbrandij *et al*., 2017). Unfortunately, so far, few data have been published on the burden of mental disorders among Syrian refugees in Turkey, the treatment gap and barriers to care. However, these data are crucial to inform the design and successful implementation of mental health programmes for Syrian refugees in Turkey.

The aim of this study is to report evidence on mental health needs and access to MHPSS care among Syrian refugees living in Istanbul, Turkey. The specific objectives are (i) to determine the estimated prevalence of symptoms of priority mental disorders among Syrian refugees in Sultanbeyli, Istanbul; (ii) to examine the mental health treatment gap and utilisation of current MHPSS services; (iii) to explore barriers to seeking and continuing care; and (iv) to investigate knowledge, perception and attitudes towards mental health among Syrian refugees.

## Methods

### Study design

A cross-sectional survey was conducted among Syrian refugees aged 18 years or over in Istanbul (Sultanbeyli district) between February and May 2018. We considered a refugee as someone who has been granted official status by the Turkish government as a person under ‘Temporary Protection’ (Republic of Turkey, 2018). Sultanbeyli is an economically deprived area of Istanbul in which over 22 000 Syrian refugees live in overcrowded conditions in rented apartment blocks (Erdogan, 2017). We undertook random sampling to select respondents from Sultanbeyli Municipality's registration system, which has an accurate register of Syrian refugees locally. The sample size calculations are included in the appendices (Appendix 1). Potential respondents were first approached by telephone and invited to attend the survey interview at a date convenient to them. Thirty Turkish Lira (equivalent to €6) were provided to respondents to compensate them for their time completing the interview. The questionnaires were administered through face-to-face interviews in a private space of a community centre in central Sultanbeyli. Interviews were conducted in Arabic by experienced researchers who were trained on the aims of the survey, ethical issues, quality standards and being sensitive to respondents' needs. Respondents and interviewers were matched by sex. Interviewers were supervised by an experienced researcher with a degree in clinical psychology. Excluded from the interview were participants currently under the influence of alcohol or drugs, non-native Arabic speakers and those with severe intellectual impairments. Interviews lasted approximately 45 min and informed consent of the respondents was sought prior to data collection. Ethical approval was obtained by the London School of Hygiene and Tropical Medicine's Institutional Review Board, and the Institutional Review Board of Istanbul Sehir University in addition to the Immigration Authority of Turkey.

### Survey questionnaire

The survey questionnaire included measures to estimate the prevalence of symptoms of depression and anxiety (Hopkins Symptoms Checklist, HSCL-25) as well as symptoms of Posttraumatic Stress Disorder Checklist (PCL-5) that have previously been used in populations affected by conflict (Mollica *et al*., 2004; Mahfoud *et al*., 2013; Roberts *et al*., 2017; Wind *et al*., 2017). We also assessed self-reported emotional problems with the following question: ‘Since you arrived in Sultanbeyli, have you ever felt feelings such as anxiety, nervousness, depression, insomnia or any other emotional or behavioural problems’? We also collected information on utilisation of mental health care services and support, barriers of seeking and continuing care, and knowledge and attitudes towards people with mental health problems. Demographic and socio-economic characteristics were obtained including sex, age, education level, marital status, living conditions, employment status and household economic situation. The survey questionnaire underwent a thorough adaptation and translation process to help ensure reliability, validity and appropriateness for the study population, based on best practice procedures (Mollica *et al*., 2004; WHO, 2018). This included translation from English into Arabic using professional translators; independent back-translation to check for accuracy, consistency and equivalence; review by Turkish, Syrian and international mental health experts for cultural relevance, content, concept consistency, clarity and understanding; as well as piloting and field-testing to further refining the instrument.

### Data analysis

We undertook a descriptive analysis. For the HSCL-25, we calculated the mean score for both depression and anxiety using the cut-off of ⩾2.10 for clinical symptoms of depression and ⩾2.00 for symptomatic anxiety as recommended by Mahfoud *et al*. who worked with a study population which is comparable with our sample of participants (language, culture and socio-economic status) (Mahfoud *et al*., 2013). For the PCL-5, we used the cut-off score of ⩾33 as the indicator of symptomatic PTSD (Blevins *et al*., 2015). The HSCL-25 and PCL-5 showed good reliability (internal consistency) in our main study sample (*N*  =  1678) with Cronbach's *α* scores of 0.87 for depression (HSCL-25), 0.86 for anxiety (HSCL-25) and 0.87 for PTSD (PCL-5), respectively.

To describe the treatment gap, we calculated the proportion of individuals who did not seek health care despite self-reporting anxiety, nervousness, depression, insomnia, or any other emotional or behavioural problem since arriving in Sultanbeyli, and who screened positive with symptoms of one or more mental disorder (either PTSD, depression or anxiety symptoms using the HSCL-25 and/or PCL-5) (Roberts *et al*., 2017). Our definition of the treatment gap is therefore based on screening questionnaires and self-perceived mental health problems, and not on a clinical diagnostic interview which would be a more accurate tool to determine treatment need. Statistical analysis was conducted using Stata 15 (StataCorp, 2015).

## Results

In total, 2865 names of Syrian refugees were drawn from the Sultanbeyli municipal registry and 1678 (59%) participated. The remaining 1187 participants either refused, missed appointments, were ineligible, could not be contacted or had died. Sample characteristics of the 1678 participants are given in Table 1. Half of the sample were women (51.6%), and the median age of the respondents was 34 years. The majority of the respondents were married (83.2%), and 9.5% of the sample were single. The highest level of education obtained was primary school for the majority of the respondents (45.9%), and around 8% of the respondents had no formal education at all. In terms of employment status, 44% of men were in regular paid work and another 23% in irregular employment or self-employed, while 92% of women described themselves as housewives. The economic situation of the household was described as bad for almost half of the respondents (43.6%). On average, respondents were in Turkey for around 3 years since being displaced from Syria. The estimated prevalence of symptoms of PTSD, depression and anxiety was 19.6, 34.7 and 36.1%, respectively (see Table 2). There was a substantial overlap in the number of people with symptoms of these three disorders, and in total, 46.9% of the sample met the criteria for symptomatic depression, anxiety or PTSD.

**Table 1.** Sample characteristics (*N*  =  1678)

Variable	*N*	%
Sex		
Male	812	48.4
Female	866	51.6
Age		
18–24	354	21.1
25–34	518	30.9
35–44	373	22.3
45–54	247	14.8
55+	182	10.9
Median (years)	34	
Marital status		
Married	1396	83. 2
Single	160	9.5
Widowed/divorced	118	7.0
Education		
No formal education	137	8.2
Primary school (1–6 years)	765	45.9
Pre-secondary (7–9 years)	520	31.2
Upper secondary (10–12 years)	130	7.8
Higher education (>12 years)	116	7.0
Employment		
Regular paid work	374	22.3
Irregular paid work	150	8.9
Self-employed	51	3.0
Unemployed/seeking work	131	7.8
Housewife	799	47.6
Retired due to old age or disability	123	7.3
Other	50	3.0
Household economic situation		
Very good	7	0.4
Good	68	4.1
Average	867	51.7
Bad	731	43.6
Number of years displaced from Syria		
<1	21	1.3
1	125	7.5
2	227	13.5
3	425	25.3
4	454	27.1
5	206	12.3
>6	107	6.4
No answer	113	6.7
Mean (years)	3.4

**Table 2.** Estimated prevalence of symptoms of PTSD (PCL-5), depression (HSCL-25), anxiety (HSCL-25) and self-reported emotional/behavioural problems (*n*  =  1678)

	Yes		No		Refused/don't know^a^	
	*n*	%	*n*	%	*n*	%
PTSD^b^	328	19.6	1318	78.6	32	1.9
Anxiety^c^	582	34.7	1077	64.2	19	1.1
Depression^c^	606	36.1	1049	62.5	23	1.4
Self-reported emotional/behavioural problems since arriving in Sultanbeyli	358	21.3	1284	76.5	36	2.2
Symptoms of PTSD, anxiety or depression and self-reported problems	249	14.8	1393	83.0	36	2.2

a Refused/don't know: participant refused response to question or did not know the answer.

b Calculated using the conventional cut-off score of ≥33.

c We defined ‘symptomatic depression’ using a cut-off score of ⩾2.10 and ‘symptomatic anxiety’ using a cut-off score ⩾2.00 (Mahfoud *et al*., 2013).

### Treatment gap and barriers to seeking care

**Fig. 1.**
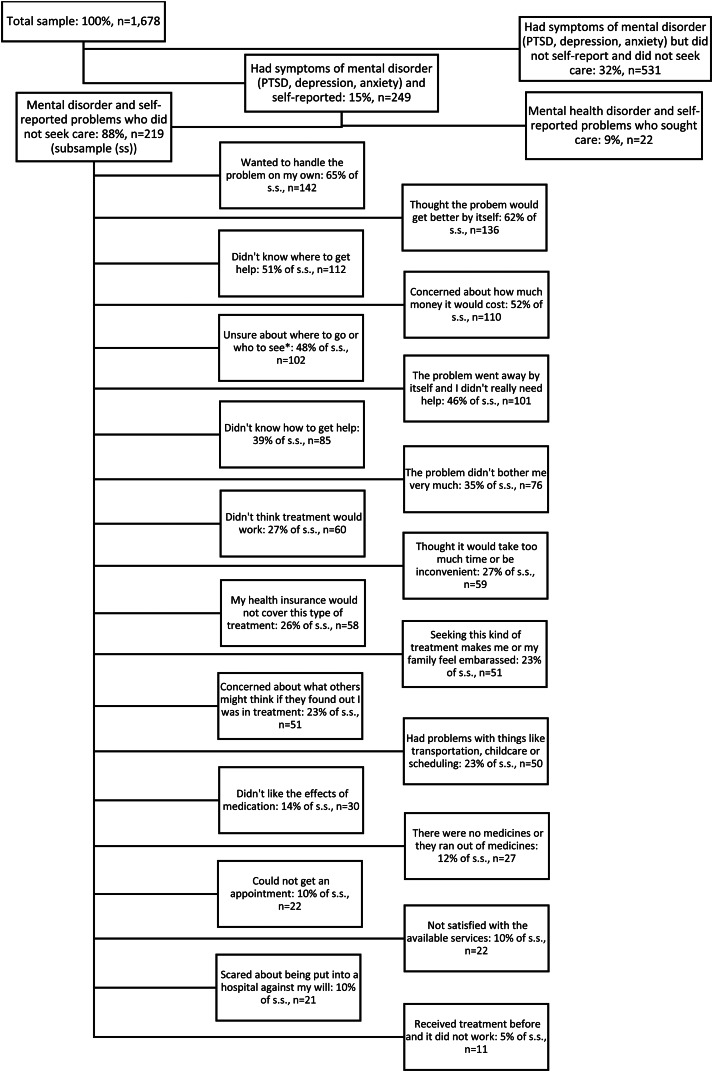
Reasons for not seeking health care in the presence of mental health symptoms (multiple answers allowed) (*N*  =  249). *Where to go or who to see  = participants did not know where to go (in terms of location and service) or who (which health professional) to see.

In total, 249 respondents (15%) screened positive for either symptoms of PTSD, depression or anxiety in our survey, and also self-reported emotional/behavioural problems since arriving in Sultanbeyli. Out of those 249 respondents, only 22 respondents (9%) sought care, and 219 respondents did not (88%). The treatment gap (i.e. the proportion of people that did not seek care) was similar for all three disorders: 89% for PTSD, 90% for anxiety and 88% for depression. The reasons for not seeking care are given in Fig. 1. Over half of the respondents wanted to handle the behavioural problem they were facing on their own, were concerned about the cost of health care or believed that time would improve symptoms. A large proportion of the respondents (*n*  =  102, 47%) were also unsure which service they should attend and did not know where and how to get help. Around one-quarter of participants (*n*  =  60, 27%) did not believe that treatment would improve symptoms, and were concerned about opportunity costs and time spent on treatment. Embarrassment to seek treatment (*n*  =  51, 23%) and the concern about what other people would think were also named as reasons for not seeking care. A small proportion of participants (*n*  =  22, 10%) mentioned unavailability of appointments, lack of medication and the fear of being put into hospital against their own will as reasons for not seeking care.

Out of the 22 respondents who sought care, 12 (55%) did not complete the full course of treatment. Respondents tended to discontinue treatment due to a desire to handle the problem on their own, and mentioned structural barriers hindering them to continuing treatment like lack of time, transportation and problems with the treatment schedule.

### Health care utilisation and sources of information on mental health

We asked all respondents (*N*  =  1678) where Syrian refugees with mental health problems usually seek help (Table 3). Over 50% of the respondents (*n*  =  907) reported government hospitals and refugee health centres as the most common places to go for mental health treatment. Family and friends (*n*  =  890, 53%) and religious leaders (*n*  =  735, 44%) were also commonly cited. Private hospitals (*n*  =  710) and local government clinics (*n*  =  700) were mentioned by around 40% of participants. Around one-third of the respondents reported NGOs (*n*  =  526, 31%) as places to seek treatment. Eight per cent (*n*  =  138) of the respondents indicated that care would not be available at all and a further 15% (*n*  =  251) did not know if members of their community sought care at any of the places presented in Table 3. The mean number of places to seek care reported by respondents was four.

**Table 3.** Places where Syrian refugees with mental health problems usually go to seek help (*n*  =  1678)^a^

	Yes		No		Refused/don't know^b^	
	*n*	%	*n*	%	*n*	%
Government hospitals	907	54.1	289	17.2	482	28.7
Family and friends	890	53.0	364	21.7	424	25.3
Refugee health centres	811	48.3	298	17.8	569	33.9
Religious leaders	735	43.8	443	26.4	500	29.8
Private hospitals	710	42.3	451	26.9	517	30.8
Local government clinic	700	41.7	408	24.3	570	34.0
NGOs/community organisations itself	526	31.4	485	28.9	667	39.8
The municipality	410	24.4	644	38.4	624	37.2
Nowhere: care is not available	138	8.2	1029	61.3	511	30.5
Other	98	5.8	813	48.5	767	45.7

a Multiple answers allowed.

b Refused/don't know: participant refused response to question or did not know the answer.

Questions were also asked about sources of information on mental health in Sultanbeyli and where information on mental health was generally obtained. Around 20% (*n*  =  348) of the respondents reported that information on mental health was provided by the Refugee and Asylum Seekers Assistance and Solidarity Association and other refugee health centres. Over 70% of the respondents indicated that health and social care institutions (*n*  =  1214, 72%) and NGOs (*n*  =  1305, 78%) operating in Sultanbeyli were not providing any information on mental health. Further information on sources of information on mental health is presented in Appendix 2.

### Knowledge, perceptions and attitudes towards mental health

**Fig. 2.**
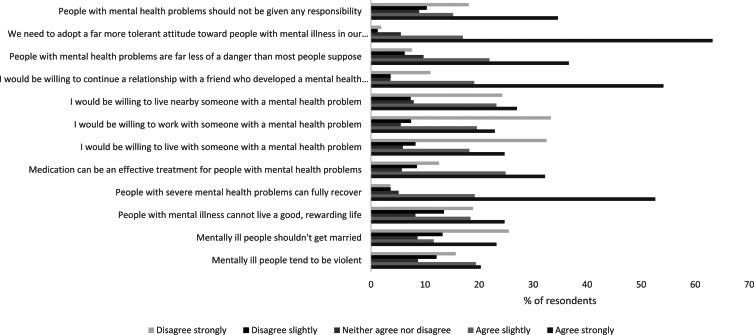
Knowledge, perceptions and attitudes towards mental health (%) (*n*  =  1678).

Out of our sample, 40% (*n*  =  667) of the respondents believed that people with mental health problems tend to be violent, and that people with mental illness cannot live a good, rewarding life (43%, *n*  =  723). Half of the respondents (50%, *n*  =  836) agreed that people with mental health problems should not be given any responsibility in life, and one-third of the respondents (34%, *n*  =  584) believed that mentally ill people should not get married. Two-thirds of the respondents believed that mentally ill people can recover (72%, *n*  =  1205), and agreed that a more tolerant attitude in society towards people with mental health problems is needed (80%, *n*  =  836). Around 70% (*n*  =  1229) of the respondents reported that they were willing to continue a relationship with a friend who developed a mental health problem. Medication was seen as an effective treatment for people with mental health problems by over half of the respondents (57%, *n*  =  958). Knowledge, perceptions and attitudes towards mental health by respondents are presented in Fig. 2 (table with data included in Appendix 3).

## Discussion

This study reports representative data on the estimated prevalence of symptoms of mental disorders as well as access and barriers to MHPSS care among Syrians refugees living in the Sultanbeyli area of Istanbul, Turkey. To the best of our knowledge, this is the first such study from Istanbul. We found high levels of symptoms of PTSD, anxiety and depression, and a high mental health treatment gap. Syrian refugees reported an array of public, private and informal community services where help for mental health problems can be sought. Despite this knowledge, less than 10% of Syrian refugees in our sample who screened positive for mental health symptoms and who self-reported problems with mental health sought care. Different barriers for not seeking care were mentioned; the most prominent ones were structural barriers (e.g. misbeliefs on the costs of treatment, opportunity costs of attending treatment) or attitudinal barriers (e.g. stigma, unfamiliarity with treatment). Syrian refugees in our sample held some negative perceptions towards mental health and people with mental health problems.

Only a few studies have been published on the estimated prevalence of mental disorder symptoms among Syrian refugees in the Middle East and Europe. These studies (also cited in the introduction) show a large variation in the prevalence of symptoms for PTSD, depression and anxiety across study countries. Inter-survey variability has been reported previously (Steel *et al*., 2009) and reflects different data collection methods, the choice of screening tools, participant recruitment setting (e.g. community *v*. primary health care), overall sample size and may as well be influenced by participant's characteristics such as length of time since being displaced. Our estimates for symptoms of depression and anxiety are similar to data reported by Acarturk who estimated the prevalence of symptoms of depression as 37.4% in adult Syrians residing in a camp near the Syrian/Turkish border (Acarturk *et al*., 2018). The prevalence of symptoms of depression and anxiety has been reported as 30–40% in other community settings, including among Syrian refugees in Greece (Poole *et al*., 2018) and Sweden (Tinghög *et al*., 2017). However, some of these studies report higher PTSD symptoms than in our sample (83% in the study by Acarturk *et al*., 2018 and 30% in Tinghög *et al*., 2017). Similarly, in a cross-sectional study conducted in a tented city in Gaziantep, Turkey, Alpak reported that 33% of Syrian refugees were diagnosed with probable PTSD evaluated by a psychiatrist (Alpak *et al*., 2015). However, lower estimates can also be found in the literature. For example, a population-based study among Syrian refugees in Erlangen, Germany (Georgiadou *et al*., 2018) reported the prevalence of symptoms of depression, anxiety and PTSD to be 14.5, 13.5 and 11.4%, respectively. Estimates from the WHO assume a lower prevalence among resettled Syrian refugees within 12 months of the emergency only (15–20% for mild and moderate symptoms of PTSD, anxiety and depression) (van Ommeren *et al*., 2005; WHO/UNHCR, 2012; Hassan *et al*., 2015; Jefee-Bahloul *et al*., 2016; CDC, 2019).

We found a large mental health treatment gap among Syrian refugees in Istanbul. There is a large mental health treatment gap for common mental disorders among the general population in almost every country (Patel *et al*., 2018), and our findings correspond to the mental health treatment gap seen among other vulnerable groups including refugees or internally displaced persons (Karam *et al*., 2008; Llosa *et al*., 2014; Chikovani *et al*., 2015; Diggle *et al*., 2017; Roberts *et al*., 2017). Mental health service utilisation among Syrian refugees in Istanbul is poor, despite the provision of mental health services in the community. Low MHPSS utilisation has been reported before (Morina and Emmelkamp, 2012; Chikovani *et al*., 2015; Roberts *et al*., 2017). A recent systematic review also highlighted that barriers to accessing mental health care for refugees and asylum seekers in Europe may include culturally-specific barriers such as language, help-seeking behaviours, lack of awareness, stigma and negative attitudes towards treatment and providers (Satinsky *et al*., 2019). Evidence is now accumulating that settled refugees often struggle to understand the health system of the host country, how to enter it and how to navigate within it (Norredam *et al*., 2006; Arie, 2015; Langlois *et al*., 2016; Mangrio and Sjogren Forss, 2017). It is for policy makers to improve the responsiveness of the mental health system, and tailor it to the needs of the patient in order to facilitate access to care (Fuhr and Roberts, 2018). This needs to be accompanied by the provision of information to refugees informing them about their entitlements to healthcare (Langlois *et al*., 2016). Treatment providers also have a share in this, and need to be trained to provide culturally relevant treatment (Satinsky *et al*., 2019), taking into account the patient's characteristics such as gender, language and their socio-economic background. There is also a need to incorporate the aspects of explanatory models of mental ill health in treatment provision which may be rooted in cultural and religious views of the patient (Hassan *et al*., 2015).

Our results highlight how a commitment to universal mental health coverage and actual provision of essential health services (Eaton and Ryan, 2017) may not result in improved population mental health for refugees if they do not seek mental health care for whatever reasons. Community-based mental health services need to be coupled with community awareness programmes (incorporating interventions to reduce stigma) and public information campaigns to encourage mental health service use. Public information campaigns may also counteract negative attitudes and perceptions by Syrian refugees towards people with mental disorders, with previous studies finding that knowledge about mental health is positively associated with better attitudes (Yamawaki *et al*., 2011). There is good evidence to suggest that community-based mental health services can be delivered by lay health care providers who can be trained to delivery psychological counselling in countries in which human resources for mental health are poor (Mutamba *et al*., 2013; van Ginneken *et al*., 2013). The ‘STRENGHTS’ (Syrian Refugees Mental Health Care System) study is currently underway in Turkey where Syrian refugees are being trained to deliver a mental health counselling intervention to their fellow refugees (Sijbrandij *et al*., 2017). Such a programme of care can help to overcome potential supply side barriers in Turkey, and could possibly increase help seeking as patients may better relate to their ‘treatment providers’.

### Limitations

First, we have drawn our sample from the Sultanbeyli Municipality's registration system which only includes data of officially registered Syrian refugees. The registration system does not include data on unregistered or undocumented Syrian refugees, who may have even higher mental health needs. Second, our estimate of the treatment gap measures contact coverage only, and does not take the quality of care into account. We do not know if the mental health treatment which Syrian refugees received is effective in improving their symptom severity, and therefore, we were unable to speculate about effectiveness coverage. Our definition of the treatment gap is based on screening questionnaires and self-perceived psychological problems which do not necessarily mean that the individual needs treatment. This is supported by the response of around one-third of the respondents who were not sufficiently bothered by the mental health problems (see Fig. 1) and therefore did not seek care. Thus, it is important to view our results as providing an upper boundary on the actual treatment gap. Notwithstanding this limitation, we believe that our findings do make a distinct contribution to the sparse evidence on the mental health treatment gap of conflict-affected populations particularly among Syrian refugees (Hendrickx *et al*., 2019; Satinsky *et al*., 2019). Third, we used a screening questionnaire to measure the symptoms of PTSD, anxiety and depression. A more in-depth diagnostic interview (which includes an assessment of functional impairment) may have resulted in more precise prevalence estimates as self-report questionnaires are associated with higher rates of mental disorders (Steel *et al*., 2009). Fourth, the cut-off score of the PCL-5, which has been used in this paper, has not been validated for Syrian refugees but has been used in other studies among male veterans in the USA (Bovin *et al*., 2016) and among conflict-affected populations in Ukraine (Roberts *et al*., 2017). For the HSCL-25, we applied the cut-off score of Mahfoud (Mahfoud *et al*., 2013) who validated his cut-off score among disadvantaged Lebanese women. Ideally, both cut-off scores of the PCL-5 and HSCL-25 should have been validated by both culture and gender. Finally, we could not engage in multivariable analysis to investigate the factors associated with accessing mental health care and the type of care individuals receive, as only a small number of respondents did access care.

## Conclusions

Our study reports the first epidemiological study on the estimated prevalence of symptoms of priority mental disorders and access to MHPSS care among Syrian refugees in Istanbul. There seems to be a large burden of PTSD, anxiety and depression among this population group, and a large mental health treatment gap. Only a small minority of Syrian refugees in need of mental health treatment sought care despite some availability of mental health services in the community. The high mental health treatment gap may be explained by barriers to seeking care which were identified in our study. Future research should explore how those barriers to help seeking can be overcome. Our study makes evident that granting refugees access to the national mental health system is only a first step that will remain insufficient if barriers to seeking care and negative attitudes towards mental ill health are not tackled. Culturally-relevant and contextually-appropriate psychological interventions need to be made accessible in community settings which overcome structural and attitudinal barriers to accessing care. This should be accompanied by community awareness interventions providing information about the functioning of the health system, mental disorders and mental health more generally to overcome the mental health treatment gap among Syrian refugees in Turkey.

## Data

Further information on the data supporting the findings of this study can be obtained from Bayard Roberts (e-mail address:Bayard.roberts@lshtm.ac.uk).

## Appendices

### Appendix 1: Sample size calculation used for study

To determine adequate sample size, the following calculation was made:

n = number of people in one group
s = standard deviation
c = 7.85 (for 80% power and 5% significance level)
d = size of the difference to be detected (0.8 SD)

Precise estimates of levels of mental disorders and associated factors could not be determined due to the limited reliable data on mental health among Syrian refugees in Turkey. Therefore, to ensure adequate power to detect conceptually important differences within a planned multivariate analysis, the following parameters and calculations were used: power = 80%; significance level = 5%; conceptually important difference in outcome scores = 0.8 standard deviation (by convention a ‘large’ difference)(Cohen, 1988); size of ‘rarest’ sub-group of respondents (for example, those experiencing a particular war or displacement experience from a particular age group) we would attempt to include in our analysis = 5%.

Using these parameters, a sample size of 314 was required to detect a difference when only 5% of the population falls into a particular sub-group of interest. Within this group, we assumed approximately 25% will have used health services (based on findings from other countries – albeit not with refugees; Roberts et al.). Therefore, the minimum sample size required is 1444, including a 15% incompletion rate.

For the sampling process, we assumed around a 50% response rate and so selected 2,865 names of Syrian refugees from the Sultanbeyli municipal registry and 1,678 (59%) participated. As a result, we were slightly above the minimum sample size required.

### Appendix 2: Sources of information provided about mental health in Sultanbeyli

**Table:**

	Yes		No		Refused/don't know	
	n	%	n	%	n	%
Internet	422	25.2	1,100	65.6	156	9.3
People talking about it	402	24.0	1,126	67.1	150	8.9
Social media	392	23.4	1,125	67.0	161	9.6
Syrian municipality (RASASA)	348	20.7	1,138	67.8	192	11.4
TV	335	20.0	1,196	71.3	147	8.8
Refugee health centres	315	18.8	1,170	69.7	193	11.5
Health and social care institution	278	16.6	1,214	72.4	186	11.1
Sultanbeyli municipality	271	16.2	1,197	71.3	210	12.5
Neighbours, family members	239	14.2	1,284	76.5	155	9.2
NGOs	154	9.2	1,305	77.8	219	13.1
Poster/leaflet	138	8.2	1,364	81.3	176	10.5
Newspaper	79	4.7	1,412	84.2	187	11.1
Magazine	76	4.5	1,412	84.2	190	11.3
Radio	70	4.2	1,426	85.0	182	10.9
Other	52	3.1	1,370	81.6	256	15.3

### Appendix 3: Knowledge, perceptions and attitudes towards mental health (%) (n=1,678)

**Table:**

	Agree strongly		Agree slightly		Neither agree nor disagree		Disagree slightly		Disagree strongly		No answer	
	n	%	n	%	n	%	n	%	n	%	n	%
Mentally ill people tend to be violent.	341	20.3	326	19.4	146	8.7	203	12.1	264	15.7	398	23.7
Mentally ill people shouldn't get married.	389	23.2	195	11.6	144	8.6	221	13.2	427	25.5	302	18.0
People with mental illness cannot live a good, rewarding life.	415	24.7	308	18.4	137	8.2	226	13.5	317	18.9	275	16.4
People with severe mental health problems can fully recover.	883	52.6	322	19.2	85	5.1	61	3.6	61	3.6	266	15.9
Medication can be an effective treatment for people with mental health problems	540	32.2	418	24.9	95	5.7	142	8.5	211	12.6	272	16.2
I would be willing to live with someone with a mental health problem.	415	24.7	306	18.2	99	5.9	138	8.2	545	32.5	175	10.4
I would be willing to work with someone with a mental health problem.	385	22.9	329	19.6	92	5.5	124	7.4	559	33.3	189	11.3
I would be willing to live nearby someone with a mental health problem.	453	27.0	390	23.2	133	7.9	122	7.3	407	24.3	173	10.3
I would be willing to continue a relationship with a friend who developed a mental health problem	908	54.1	321	19.1	61	3.6	60	3.6	184	11.0	144	8.6
People with mental health problems are far less of a danger than most people suppose	614	36.6	367	21.9	162	9.7	104	6.2	127	7.6	304	18.1
We need to adopt a far more tolerant attitude toward people with mental illness in our society.	1,060	63.2	285	17.0	93	5.5	20	1.2	32	1.9	188	11.2
People with mental health problems should not be given any responsibility.	581	34.6	255	15.2	149	8.9	172	10.3	303	18.1	218	13.0
